# Case report: Whole exome sequencing and genome-wide methylation profiling of Czech dysplasia in a Chinese pedigree

**DOI:** 10.3389/fmed.2023.1244888

**Published:** 2023-11-02

**Authors:** Mengfei Zhao, Runrun Zhang, Cen Chang, Yehua Jin, Lingxia Xu, Shicheng Guo, Steven Schrodi, Yong He, Dongyi He

**Affiliations:** ^1^Department of Rheumatology, Shanghai Guanghua Hospital, Shanghai University of Traditional Chinese Medicine, Shanghai, China; ^2^High Dependency Unit, Shanghai NO.3 Rehabilitation Hospital, Shanghai, China; ^3^The Second Clinical Medical College, Zhejiang Chinese Medical University, Hangzhou, China; ^4^Department of Rheumatology, The Second Affiliated Hospital of Zhejiang Chinese Medical University, Hangzhou, China; ^5^Arthritis Institute of Integrated Traditional and Western Medicine, Shanghai Chinese Medicine Research Institute, Shanghai, China; ^6^Department of Medical Genetics, School of Medicine and Public Health, University of Wisconsin-Madison, Madison, WI, United States; ^7^Department of Orthopedics, Shanghai Guanghua Hospital, Shanghai University of Traditional Chinese Medicine, Shanghai, China

**Keywords:** Czech dysplasia, *COL2A1*, whole exome-sequencing, mutation, Chinese pedigree

## Abstract

**Background:**

Czech dysplasia is a rare skeletal disorder with symptomatology including platyspondyly, brachydactyly of the third and fourth toes, and early-onset progressive pseudorheumatoid arthritis. The disorder segregates in an autosomal dominant fashion. A specific missense mutation (R275C, c.823C > T) in exon 13 of the *COL2A1* gene has been identified in German and Japanese families.

**Case summary:**

We present the case of a Chinese woman diagnosed with Czech dysplasia (proband) who carried a variant in the *COL2A1* gene. Whole-exome sequencing (WES) identified the *COL2A1* missense mutation (R275C, c.823C > T) in close relatives of the proband who also exhibited the same disorder.

**Conclusion:**

This study is a thorough clinical and physiological description of Czech dysplasia in a Chinese patient.

## Introduction

Czech dysplasia (OMIM 609162), also known as spondyloepiphyseal dysplasia with metatarsal shortening (1), is an autosomal dominant type II collagenopathy characterized by early-onset progressive pseudorheumatoid, platyspondyly, brachydactyly of the third and fourth toes, and normal ocular anatomy and height. Initially referred to as Dominantly Inherited Psudorheumatic Arthritis, it was later named Czech dysplasia in 2004. Because all the patients were Caucasians and all came from the Czech Republic, not all the members experienced the pain associated with weather conditions (2). In 2007, a specific missense mutation (R275C, c.823C > T) in the triple helical domain of the *COL2A1* gene was identified in five unrelated patients with Czech dysplasia (3). In 2008, a large German family diagnosed with R275C Czech dysplasia was found to have hearing loss in all affected relatives (4). In 2009, a case of Czech dysplasia in a family was reported in Japan (5). Researchers studied these cases of familial Czech dysplasia and found that it was caused by missense mutations in the triple helix domain of the *COL2A1* gene (R275C, c.823C > T) (4, 5). In the Chinese population, reports of Czech dysplasia are rare. In clinical practice, when encountering patients with symptoms suggestive of Czech dysplasia, whole exome sequencing was performed on the peripheral blood of the patients to confirm the diagnosis.

## Case presentation

### Patient presentation

**Figure 1:**
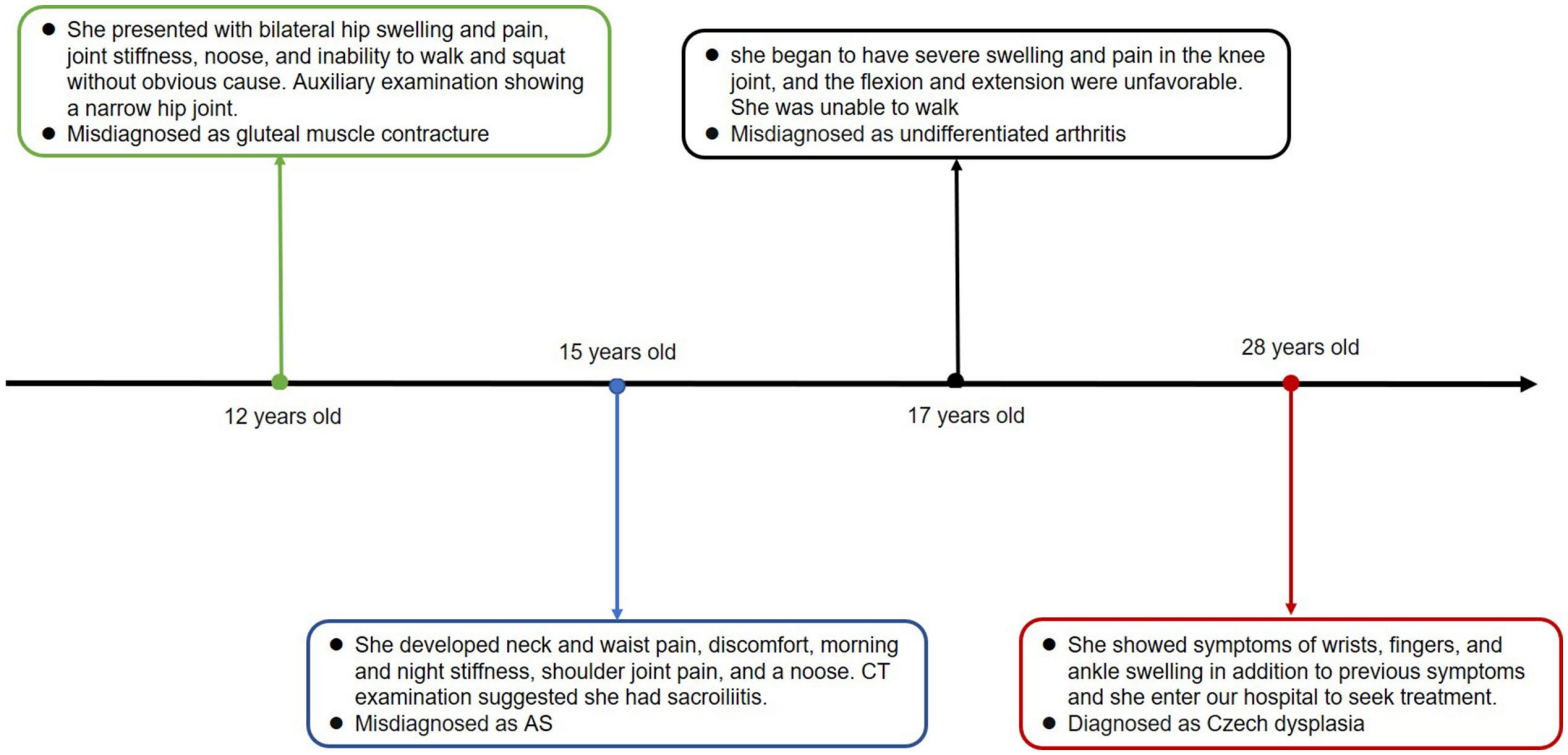
Timeline of the case report.

We present the case of a 28-year-old woman who was admitted to the hospital with a 16 years history of swelling and pain in both lower extremities. When she was 12, she experienced bilateral hip swelling and pain, joint stiffness, an inability to walk and squat, and severe pain. She was diagnosed with gluteal muscle contracture, with an auxiliary examination showing a narrow hip joint. The symptoms were gradually relieved with the help of traditional Chinese medicine and acupuncture treatment. At the age of 15, the patient began experiencing neck and waist pain, discomfort, morning and night stiffness, along with pain in the shoulder joints. A CT examination of the patient indicated sacroiliitis, leading to a misdiagnosis of ankylosing spondylitis (AS). Despite undergoing treatment with methotrexate, etanercept, hormone therapy, leflunomide, and folic acid for over a year, her symptoms did not show improvement. At the age of 17, the patient began to suffer from severe swelling and pain in the knee joint, and the poor flexion and extension indicated highly compromised mobility. She was unable to walk and was diagnosed with undifferentiated arthritis at another hospital. Subsequently, she underwent treatment with anti-inflammatory painkillers and medication, including nutritional joint medicine, which provided relief. However, the symptoms recurred later. In December 2018, the patient exhibited symptoms of swelling in the wrists, fingers, and ankles, in addition to previous symptoms, prompting her to seek treatment at our hospital. The timeline of the case report is depicted in Figure 1.

### Physical examinations

**Figure 2:**
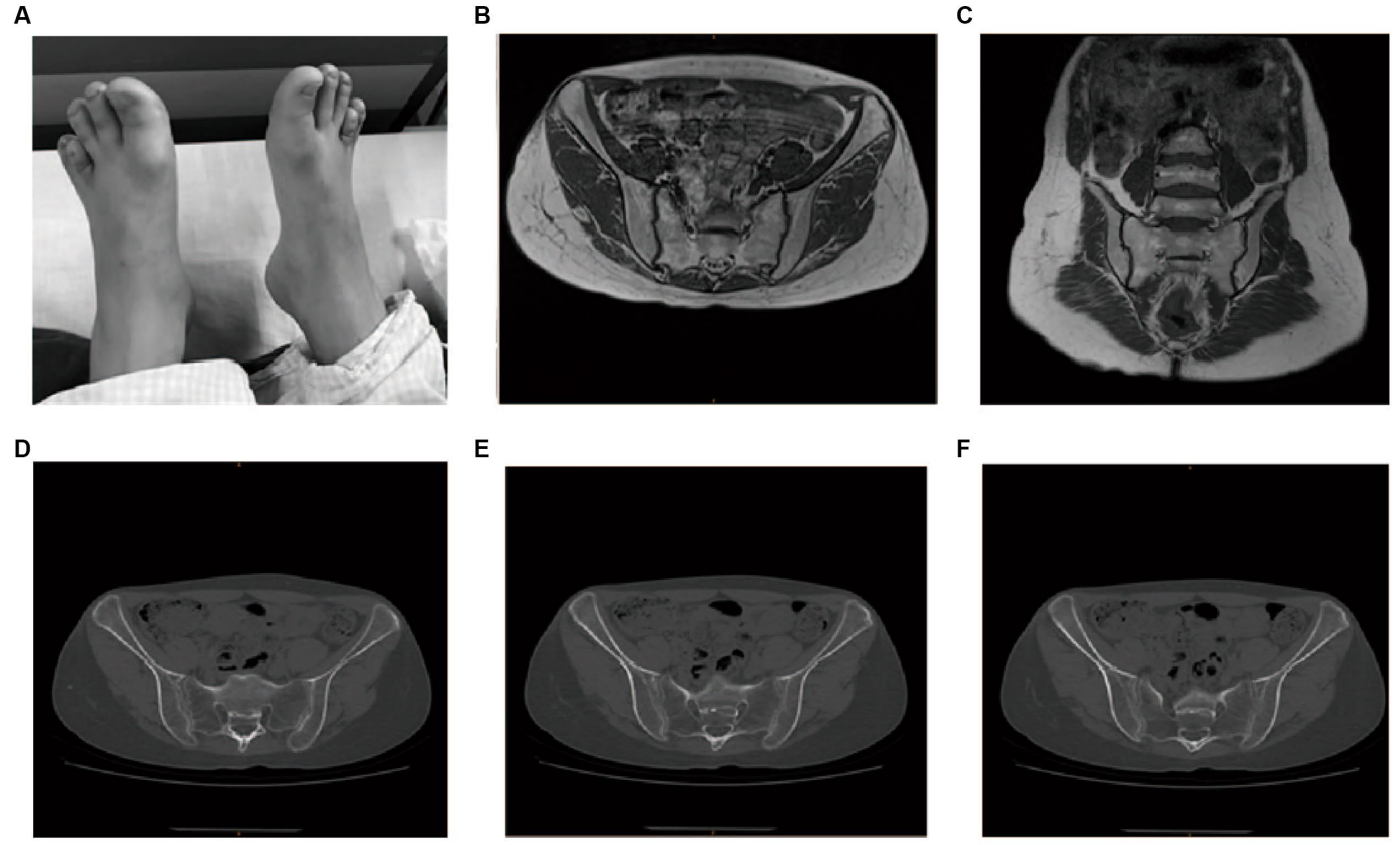
Physical examination, MRI, and CT imaging of the proband. **(A)** Bilateral short fourth metatarsal; **(B,C)** MRI for the sacroiliac joint; **(D–F)** CT for the sacroiliac joint.

The left shoulder joint exhibited slight swelling and tenderness at the condyle. Activities involving lifting and reaching were limited. The lower limbs were of equal length, with limited internal and external rotation activities. Patrick’s test was positive. There was swelling in the knee joints, local tenderness points, extreme flexion pain, palpation of the sputum, and obvious nodules or clumps. The range of motion was 0–120°, and the inner and outer grinding experiments were positive. The lateral stress was negative, the lower limb muscle strength was V, the bilateral fourth metatarsal was short (Figure 2A), and the bilateral dorsal artery could be reached.

### Laboratory findings

The patient’s routine blood tests, C-reactive protein (CRP), erythrocyte sedimentation rate (ESR), and liver and kidney functional electrolytes were all normal. All the autoimmune antibody tests, including RF (IgM, IgA, and IgG), IgG, IgA, IgM, C3, C4, anti-DNA-ds, DNA-ss, anti-CCP, HLA-B27, and ANA returned negative. Magnetic resonance imaging (MRI) of the sacroiliac joint indicated that these joints were properly aligned, with no stenosis in joint space and a smooth joint surface (Figures 2B,C). Computed tomography of the sacroiliac joint indicated that these joints were properly aligned with a slightly hyperplastic joint edge and no narrowing of the joint space (Figures 2D–F).

### Imaging data

The x-rays of both knees revealed medial and lateral stenosis of the femoral condyle of the knee joint, hyperosteogeny of the joint margin, lip-like changes, and multiple nodular-like free bodies in the knee joint capsule. In round or oval high-density nodules, the nodule density was less uniform, and the central density was lower than the circumference (Supplementary Figure S1A). The CT examination of the right knee revealed narrowed medial and lateral space in the knee joint, and multiple nodular bone densities were observed in the joint capsule, with a maximum diameter of 12 mm (Supplementary Figure S1B).

The MRI examination of the right knee joint revealed that there was no obvious stenosis in the medial and lateral spaces of the joint. There was no obvious bone marrow edema in the bones. There were multiple granular osteoids on the anterior and posterior margins of the joint. The diameter of the joint was about 5–16 mm. In the MRI image, the free body displayed an upper part with a T1 phase density close to the muscle mass, while the T2 phase exhibited a low signal density area that corresponds to the calcification image displayed by the CT examination (Supplementary Figure S2).

### Whole exome sequencing and Infinium methylation EPIC BeadChip analysis

**Figure 3:**
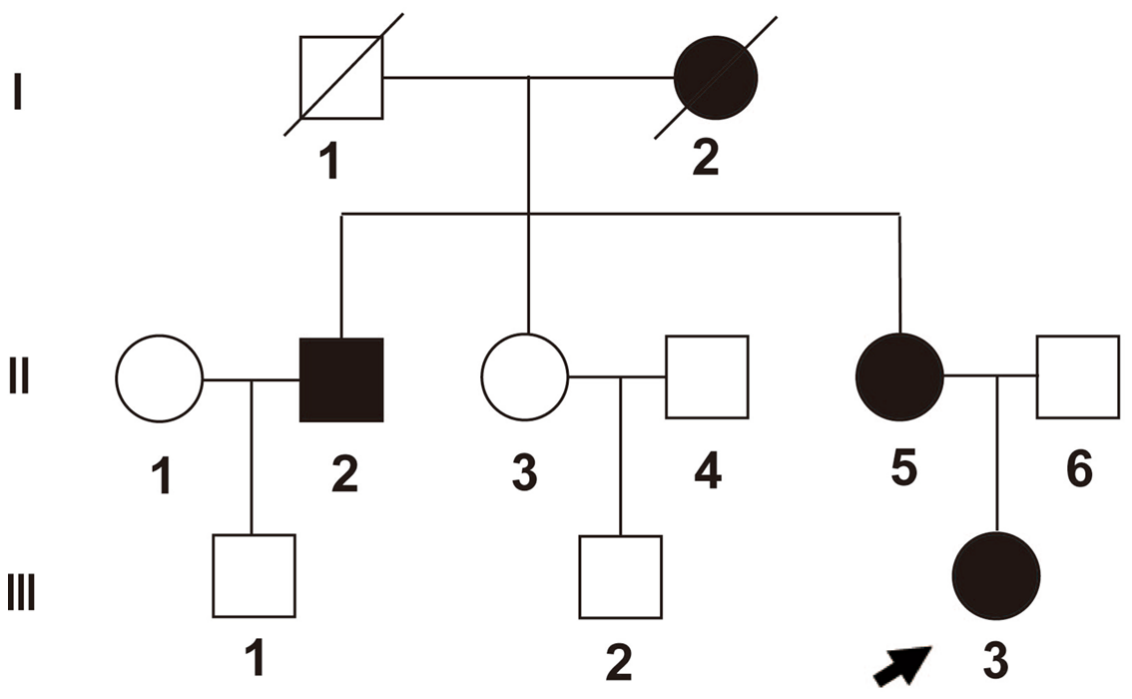
Pedigree of the family. Proband (III-3) is identified with an arrow. Box shapes represent men, circle shapes represent women; black shapes represent affected family members, and white shapes represent healthy family members.

We realized that this was a heritable genetic disorder, as multiple members of the patient’s family, such as the grandmother, uncle, and mother of the patient, showed similar symptoms. The patient’s grandmother suffered from bilateral hip pain and limited movement at almost 30 years of age. She could not walk at the age of 40 and died when she was 88. The patient’s uncle developed these symptoms when he was almost 50 years old. The patient’s mother developed the symptoms at the age of 35 (Figure 3 and Supplementary Table S1). To identify the pathogenic gene for this pedigree, we applied whole-exome sequencing to six members of the pedigree itself, which included three cases (II2, II5, and III3) and three controls (II6, III1, and III2).

**Figure 4:**
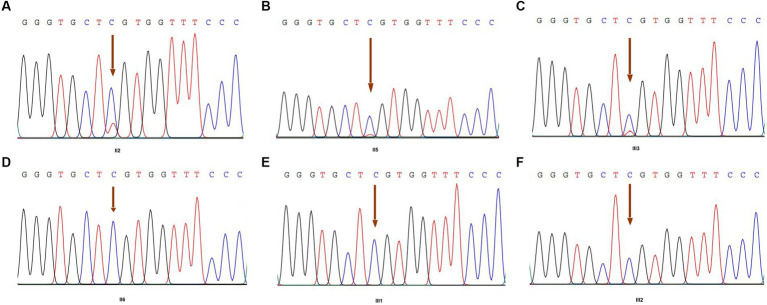
*COL2A1* variant by Sanger sequencing of family members. **(A)** The result of member II2; **(B)** the result of member II5; **(C)** the result of member III3 (proband); **(D)** the result of member II6; **(E)** the result of member III1; and **(F)** the result of member III2. Arrowheads indicate the heterozygous C to T transition at nucleotide 823 (c.823C > T), which predicts an arginine to cysteine missense mutation at codon 275 (R275C).

We performed high-depth exome-sequencing (overall average depth = 146x, ranging from 118x to 164x) for each sample, in which each has approximately 75 million 150 bp pair-end mapped reads. We identified that more than 99.5% of target genomic regions have at least 10x sequencing depth for calling variants. Overall, we detected 50,650 variants within target regions and 123,120 within 150 bp flank regions, but 143,496 SNPs and 30,274 indels. We found 1,146, 1,136, and 5,417 very high, high, and medium priority SNPs, respectively. Among these variants, there are 17,732 non-synonymous SNVs, 18,420 synonymous SNVs, 438 frameshift variants, and 195 stop-loss or stop-gap variants. After thorough manual filtering and review, the particular missense mutation (R275C, c.823C > T) in exon 13 of the *COL2A1* gene was found in our data (Figure 4). After examining clinical symptoms, we further determined that this pedigree disease was Czech dysplasia.

We performed Infinium Methylation EPIC BeadChip analysis on the samples to further explore the generation of variants. A total of seven *COL2A1* methylation sites were detected. Unfortunately, the FDR values of these sites were all greater than 0.05.

## Discussion

*COL2A1* encodes for the collagen type II alpha 1 chain (6) found in human cartilage and eye vitreous on chromosome 12 (7, 8). Type II collagen plays a vital role in endochondral bone formation and growth (9). Mutations in the *COL2A1* gene affect endochondral ossification and linear bone growth with structurally abnormal type II collagen (10). Interestingly, there is a wide spectrum of phenotypes that have been attributed to different *COL2A1* variants, including achondroplasia, early-onset familial osteoarthritis, congenital vertebral dysplasia, Stickler syndrome, Kniest dysplasia, and Strudwick congenital spine dysplasia (7, 11). Recently, a Chinses study found that the p.Arg275Cys mutation in the *COL2A1* gene often led to toe malformation and was considered a mutational hotspot for Czech dysplasia (10). This is the first report of Czech dysplasia in the Han Chinese population and therefore expands our epidemiological knowledge of the disorder. In addition, several other disorders, such as achondrogenesis type II, Kniest dysplasia, Legg-Calve-Perthes disease, spondyloepiphyseal dysplasia, and Stickler syndrome, are caused by *COL2A1* variants (occurring in different regions of the gene), and therefore, understanding the molecular pathophysiology and clinical presentation of the patients studied in this work can potentially lead to insights for those additional type II collagenopathies. In addition, understanding rare variants with linked phenotypes can aid in the delineation of *COL2A1* function, which carries importance for common complex diseases as well, given that osteoarthritis susceptibility has been associated with *COL2A1* polymorphisms (12, 13).

In this study, we applied whole-exome sequencing to a pedigree and found that the familial disease reported in this study is caused by non-synonymous variants in exon 13 of the protein encoded by *COL2A1*. Combined with its clinical symptoms, we confirmed the diagnosis of Czech dysplasia. The specific missense mutation (R275C, c.823C > T) in exon 13 of *COL2A1* was also found in our study. Variants in the *COL2A1* gene may cause dysplasia of the tibia and, to some extent, explain why the fourth metatarsal is shorter in patients in this study.

Swelling, pain, and stiffness occur in multiple joints of the patient (III3), which is similar to the clinical presentation of early-onset progressive pseudorheumatoid arthritis. Laboratory tests on the proband (III3) showed no significant abnormalities and included CRP, ESR, RF (IgM, IgA, IgG), IgG, IgA, IgM, C3, C4, anti-DNA-ds, DNA-ss, anti-CCP. HLA-B27, and ANA. The CT and MRI results of the sacroiliac joint showed that there was no evidence of lesions in his bilateral sacroiliac joints. X-ray results of both knees showed medial and lateral femoral condyle stenosis, osteogenic hyperplasia of the articular margin, labial changes, and multiple nodular-free bodies in the knee capsule. CT results of the right knee showed that the medial and lateral spaces were narrowed, and multiple nodular bone densities could be observed in the joint capsule. An MRI of the right knee revealed multiple granular bone shapes at the anterior and posterior margins of the joint.

Our patients who were treated with NSAIDs, disease-modifying drugs (e.g., methotrexate, leflunomide), and biologics (etanercept) experienced some brief relief of their symptoms but then relapsed. It has been reported that pain and joint range of motion improved in patients with type II collagen after immunomodulatory therapy (1). However, this patient experienced significant improvement in symptoms after surgical therapy. Clinicians should be aware of short third and fourth toes and sensorineural hearing loss in patients with early-onset progressive pseudorheumatoid arthritis, especially when inflammatory markers are absent and x-rays show osteoarthritis. Czech dysplasia (OMIM 609162) is an autosomal dominant type II collagenopathy that can be disabling and negatively affect the quality of life; thus, prenatal diagnosis and genetic counseling are recommended for family planning and to detect other family members who may be carriers.

The mutation discovered in this family is a missense variant at amino acid 275 that replaces the arginine residue with a cysteine. This modification occurs within the pro-alpha1(II) chain of the polypeptide, which may modify the production of procollagen, thereby driving cartilage pathology. The site of this variant exhibits substantial conservation across vertebrates, as measured by the Basewise conservation PhyloP score (score = 7.43). Although speculative, *COL2A1* is known to be expressed in the prostate, stomach, testis, and intestinal tissues, so the variant may also have subclinical effects in these tissues within patients.

In conclusion, our study provides further evidence for the independent occurrence of Czech dysplasia among the population. The diagnosis of Czech dysplasia involves physical examination, medical history analysis, imaging studies such as x-rays, and genetic testing. Treatment options are limited, and management typically focuses on alleviating symptoms and addressing complications. This may involve physical therapy, drug therapy, and surgical treatment to improve mobility and quality of life. As Czech dysplasia is a genetic disorder, there is no known cure. However, ongoing research in the fields of genetics and orthopedics may provide better insights into the condition and potentially lead to more targeted treatment options in the future.

## Data availability statement

The original contributions presented in the study are publicly available. This data can be found in NCBI’s SRA data, accession number PRJNA1028766, https://www.ncbi.nlm.nih.gov/sra/PRJNA1028766.

## Ethics statement

The studies involving humans were approved by institutional review board (IRB) of Shanghai Guanghua Hospital (IRB: HS17GX254-2017). The studies were conducted in accordance with the local legislation and institutional requirements. The participants provided their written informed consent to participate in this study. Written informed consent was obtained from the individual(s) for the publication of any potentially identifiable images or data included in this article.

## Author contributions

DH, YH, and SG contributed to the conception, design, and final approval of the submitted version. YJ, LX, and CC contributed to the analysis of sequencing data and statistical analysis. The final manuscript was completed by RZ and MZ. All authors contributed to the article and approved the submitted version.
