# Supplementary figures and images for: Case report: Whole exome sequencing and genome-wide methylation profiling of Czech dysplasia in a Chinese pedigree
Source: Front Med (Lausanne). 2023 Nov 2;10:1244888. doi: 10.3389/fmed.2023.1244888 (PMC10652562; doi:10.3389/fmed.2023.1244888)

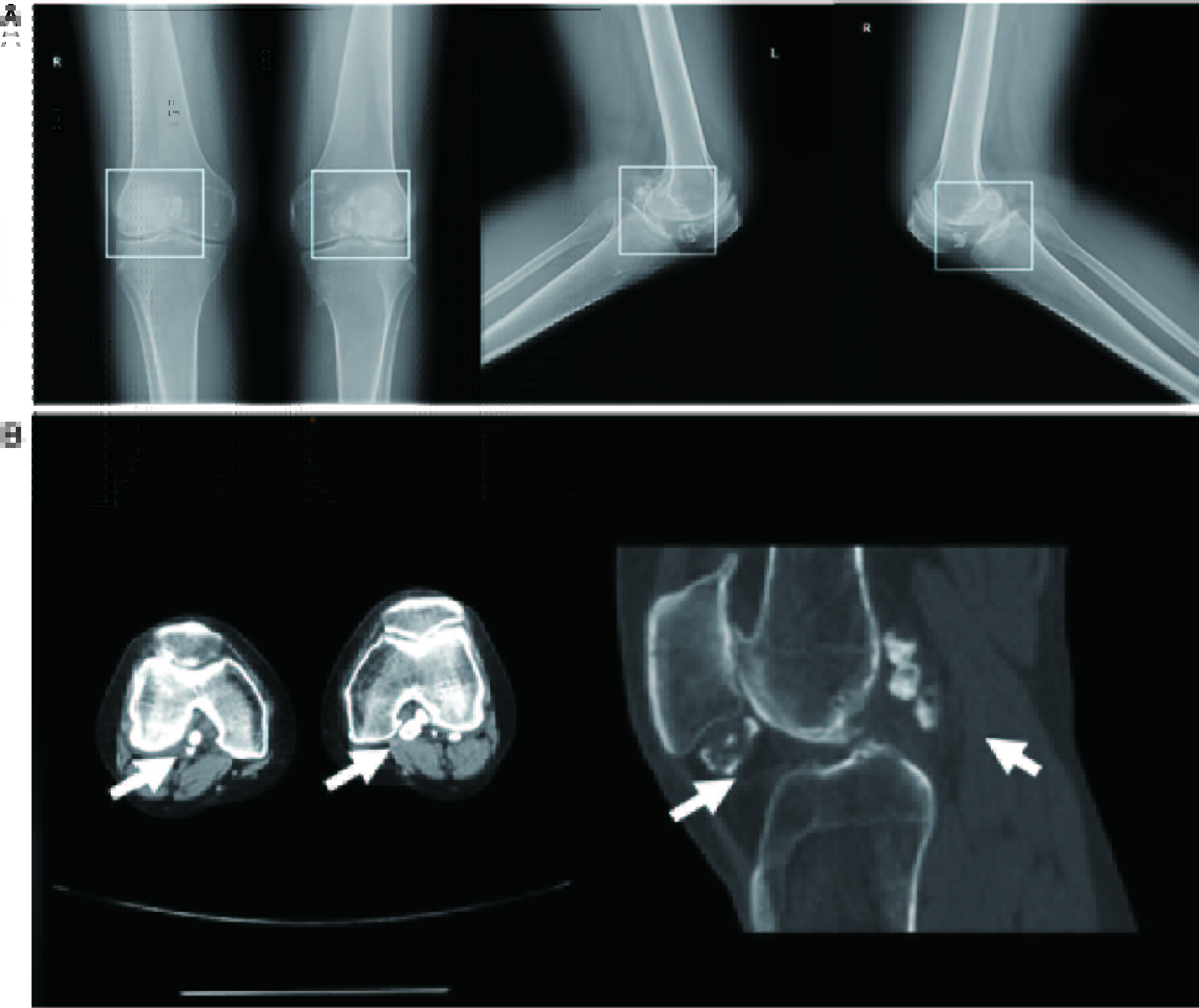

Supplement: Supplementary file 1 [file Image_1.JPEG]

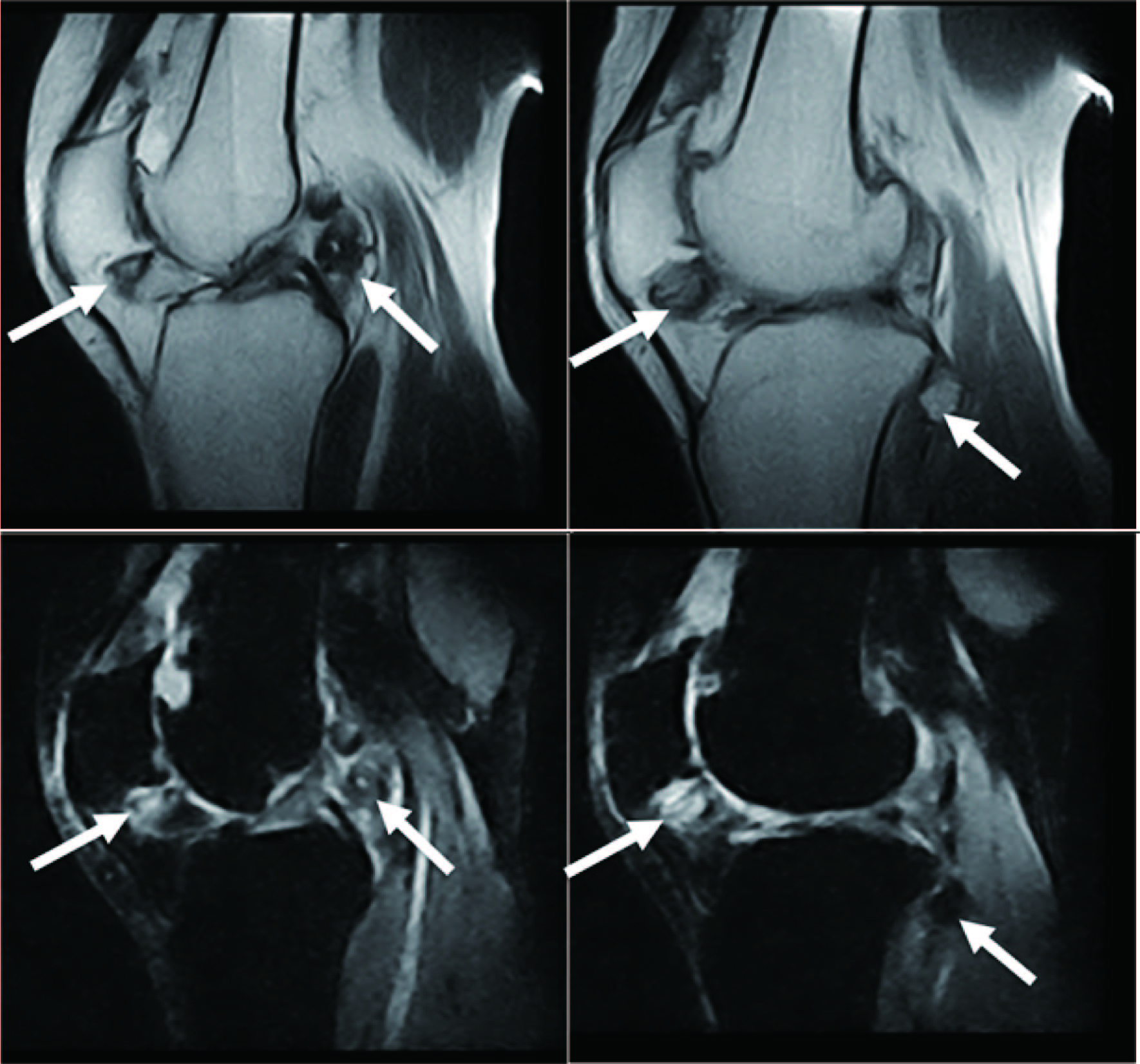

Supplement: Supplementary file 2 [file Image_2.JPEG]
