# Supplementary material for: Case report: Whole exome sequencing and genome-wide methylation profiling of Czech dysplasia in a Chinese pedigree
Source: Front Med (Lausanne). 2023 Nov 2;10:1244888. doi: 10.3389/fmed.2023.1244888 (PMC10652562; doi:10.3389/fmed.2023.1244888)
Supplement: Supplementary file 3 [file Table_1.docx]

# Supplementary Table 1. Pedigree basic information

|  | Current Age | Onset of age | Onset Joint | Affected Joint |
| --- | --- | --- | --- | --- |
| I 2 | Died aged 88 | 30 | hip joint | Hip, Knee |
| II 2 | 63 | 50 | hip joint | Hip, Knee |
| II 5 | 54 | 35 | hip joint | Hip, Shoulder |
| III 3 | 28 | 13 | hip joint | Hip, Knee, Shoulder |
